# Optilume® Balloon Dilatation for Urethral Stricture Disease: An Institutional Review of Outcomes for Multiple Stricture Aetiologies and Characteristics

**DOI:** 10.7759/cureus.91919

**Published:** 2025-09-09

**Authors:** Rustam Karanjia, Andrew Pineda-Turner, Isabella Watts, Danielle Whiting, Andrew Chetwood

**Affiliations:** 1 Urology, Frimley Health National Health Service (NHS) Foundation Trust, Frimley, GBR; 2 Public Health, Imperial College School of Public Health, London, GBR

**Keywords:** endo urology, optilume, urethral stricture (us), urethra stricture, urethroplasty

## Abstract

Introduction

Optilume^® ^balloon dilatation has shown encouraging results for men with urethral stricture disease. The Re-establishing Flow Via Drug Coated Balloon for the Treatment of Urethral Stricture Disease (ROBUST) trials demonstrated superior treatment efficacy compared to conventional endoscopic management, although they had very strict inclusion criteria. We present our patient case series, with their outcomes, to demonstrate its efficacy across all patient and stricture demographics.

Methods

Between February 2022 and December 2024, patients who underwent Optilume^®^ balloon dilatation for urethral strictures and bladder neck stenoses were analysed. Demographics, stricture characteristics, prior treatments and post-operative outcomes were gathered. The primary outcome was the need for reintervention and/or restarting self-dilatation. Cox regression analysis was performed to generate hazard ratios (HRs), 95% confidence intervals (95%CI) and identify associations with failure.

Results

Thirty-eight patients were identified with a median follow-up of 22.9 months (range 6.1-40.5). Median patient age was 64 years (range 21-86 years), stricture diameter 6Fr (range 3-16Fr), stricture length 3 cm (range 1-5) and prior number of treatments two (range 0-12). Seventeen (44%) were idiopathic strictures, 12 (32%) iatrogenic, seven (19%) radiotherapy/brachytherapy induced and two (5%) bladder neck contractures. At the time of reporting, 31/38 (82%) patients were reintervention free. There was no association with time to failure for age (HR 0.98, 95% confidence interval (CI) 0.91-1.04, *p*=0.46), stricture length (HR 0.94, 95%CI 0.38-2.31, *p*=0.89), lumen size (HR 0.89, 95%CI 0.61-1.29, *p=*0.53), number of prior treatments (HR 1.13, 95%CI 0.86-1.49, *p*=0.39) or any stricture aetiology.

Conclusions

Optilume^® ^balloon dilatation produced excellent outcomes for the treatment of urethral strictures across multiple stricture aetiologies, characteristics, and patient demographics. It may have curative potential for patients who are unfit or wish to avoid the morbidity of urethroplasty.

## Introduction

Urethral stricture disease confers significant morbidity to both patients and healthcare systems. It affects approximately 0.6% of men throughout their lifetime and is increasingly common with age [1,2]. In 2016, the National Health Service (NHS) spent over £16 million treating patients with urethral stricture disease, an increase of over £6 million from 2010 [3,4]. This is likely due to an ageing population and the increased prevalence of iatrogenic strictures, which now contribute to half of all strictures in the developed world [5,6]. Strictures are also more commonly seen in younger men following an increased recognition and treatment of both balanitis xerotica obliterans (BXO) and hypospadias disease in childhood [2,7].

Approximately half of all patients will have a recurrence following initial dilatation and it is a near certainty that further endoscopic management will result in treatment failure [2,8-10]. Urethroplasty is currently the only curative treatment available for recurrent bulbar strictures with published success rates of up to 85% [11]. However, urethroplasty can confer significant morbidity, which precludes its use in unfit patients, and can be unappealing to many others, including younger men who are concerned about the risks of sexual dysfunction [12].

The initial Re-establishing Flow Via Drug-Coated Balloon for the Treatment of Urethral Stricture Disease (ROBUST) trials of Optilume® balloon dilatation for treating recurrent bulbar urethral strictures showed excellent results, with 73% of patients remaining reintervention free at four years in the randomised control trial ROBUST III [13]. However, there remains a paucity of published data outside of clinical trial settings, which had strict inclusion criteria, limiting its applicability to the general population. We therefore aimed to assess the efficacy of Optilume® in treating recurrent bulbar urethral strictures across a wide range of patient demographics, stricture characteristics, and aetiologies.

## Materials and methods

Between February 2022 and December 2024, patients who underwent Optilume® balloon dilatation for recurrent bulbar urethral strictures were retrospectively analysed. Patients were initially selected for Optilume® if they had recurrent bulbar strictures <3 cm on either pre-operative urethrogram or previous operative findings. All patients were extensively counselled by a urethral reconstructive surgeon regarding other treatment options, including urethroplasty if suitable. As the technique was developed and safety was confirmed, indications were extended to include recurrent bladder neck stenoses. Patients were excluded from analysis if there was data missing, less than six months follow up or if they had undergone previous Optilume® balloon dilatation.

Aetiologies were divided into idiopathic, iatrogenic, radiotherapy (EBRT) or brachytherapy (BT) induced strictures, or bladder neck contractures. Patient demographics, stricture characteristics, number of prior treatments and post-operative outcomes were gathered. Significant co-morbidities were also included (defined as a history of myocardial infarction, heart failure, peripheral vascular disease, cerebrovascular event (CVA), dementia, chronic obstructive pulmonary disease (COPD), peptic ulcer disease, hemiplegia, moderate to severe chronic kidney disease (CKD), leukaemia or lymphoma).​ All patients had clinic appointment follow-up at six months with further appointments annually thereafter. All patients had direct contact access to the urology team for earlier review at any point if symptoms deteriorated.

The primary outcome was the need for reintervention or the need to restart self-dilatation. Secondary outcomes were immediate complications (Clavian-Dindo ≥2). Time-to-event analysis was performed using Cox proportional hazards regression to identify patient demographics and stricture characteristics associated with time to failure. Hazard ratios (HRs) and corresponding 95% confidence intervals (95%CI) were calculated to quantify these associations. Due to small sample size, subgroup analysis to identify associations between stricture aetiology and other patient demographics with the likelihood of failure were evaluated using Fisher’s Exact Test. All statistical analysis was performed using R statistical computing software (R version 4.4.2 (2024-10-31 ucrt), R Foundation for Statistical Computing, Vienna, Austria).

**Figure 1:**
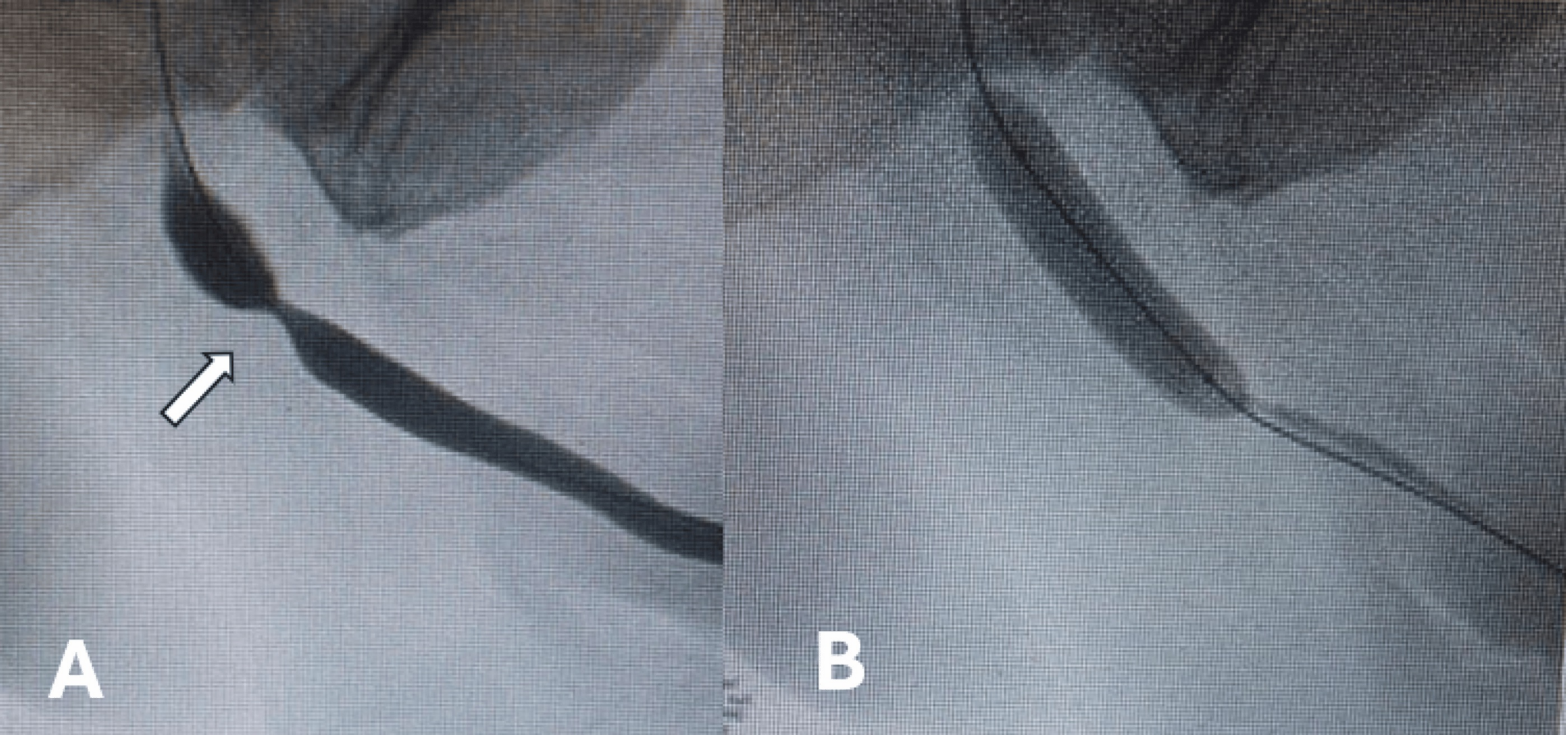
A retrograde urethrogram (A) shows a short bulbar urethral stricture (arrowed) and dilated using Optilume® balloon with contrast filling (B).

All dilatations were initially performed under general anaesthesia or sedation in lithotomy position using a rigid cystoscope. As experience improved, we switched to a supine position and a flexible cystoscope to reduce operative time and also included fluoroscopic imaging for an intra-operative urethrogram. In this technique, the patient was positioned supine with the hip flexed in traditional urethrogram position. An on-table urethrogram was then performed to identify the length and location of the stricture. A flexible cystoscope was passed for endoscopic examination of the stricture and to place a 0.035” hydrophilic guidewire into the bladder under direct vision. The cystoscope was removed and a 30 French (Fr) Optilume® balloon positioned under fluoroscopic guidance to ensure the stricture was treated in its entirety (Figure 1). The balloon was inflated at a pressure of 10 atm for seven minutes, deflated and removed. The bladder was then either drained with an in/out 12Fr catheter or a two way catheter was left in situ for three to five days before trial without catheter (TWOC) at the surgeons’ discretion.

## Results

A total of 41 patients underwent Optilume® balloon dilatation during this time. Three patients were excluded: one was missing from follow-up and two had not yet reached the six-month follow-up period. Thirty-eight patients were therefore included with median follow-up of 22.9 months (range 6.1-40.5 months). Median patient age was 64 years (range 21-86 years), of which 11 (29%) had at least one significant co-morbidity. A total of 4/38 (10%) had type 2 diabetes mellitus (T2DM) and 4/38 (10%) were on either a direct oral anticoagulant (DOAC) or clopidogrel. Median stricture diameter was 6 Fr (range 3-16 Fr), stricture length 3 cm (range 1-5 cm) and prior number of treatments was two (range 0-12). Seventeen (44%) were idiopathic strictures, 12 (32%) were iatrogenic, seven (19%) were radiotherapy (EBRT) or brachytherapy (BT) induced and two (5%) were bladder neck contractures. Two patients with idiopathic strictures had been previously treated with urethroplasty. A full list of patient demographics is included in Table 1.

**Table 1:** Full list of patient demographics and stricture characteristics

Patient characteristics (n=38)	Median (range or %)
Age (years)	62 (21–86)
Stricture aetiology	
Idiopathic+	17 (44)
Iatrogenic	12 (32)
Radiotherapy/brachytherapy	7 (19)
Bladder neck contracture	2 (5)
Previous stricture treatments	
Nil	1 (3)
Optical urethrotomy or dilatation only	35 (92)
Urethroplasty	2 (5)
Prior treatment number	2 (0–12)
Stricture length (cm)	3 (0–5)
Stricture diameter (Fr)	6 (3–16)
Diabetes	
Type 2 Diabetes Mellitus	4 (10)
No	34 (90)
Anticoagulant*	
Yes	3 (8)
No	35 (92)
Antiplatelet**	
Yes	1 (3)
No	37 (97)
Catheter post-op	
Yes	6 (16)
No	32 (84)
Significant co-morbidity***	
None	27 (71)
One	8 (21)
Two	2 (5)
Three	1 (3)
^+ ^Included two patients post urethroplasty.	
* Direct oral anticoagulant (DOAC) or warfarin.	
** Clopidogrel or ticagrelor.	
*** Defined as a history of myocardial infarction, heart failure, peripheral vascular disease, cerebrovascular event, dementia, chronic obstructive pulmonary disease, peptic ulcer disease, hemiplegia, moderate to severe chronic kidney disease, leukaemia or lymphoma.	

**Figure 2:**
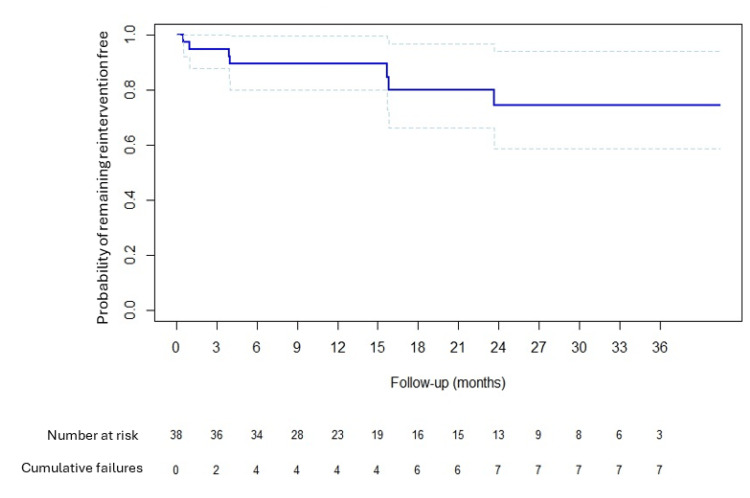
Kaplan-Meier curve, with 95% confidence intervals, demonstrating the likelihood of remaining reintervention free against time. Number at risk: number of patients who have reached this length of follow-up (months) and who have not been censored from analysis due to treatment failure. Cumulative failures: the total number of patients who failed Optilume treatment and the times at which they failed.

At the time of reporting, 31/38 (82%) patients were reintervention-free (Figure 2). For the seven patients who required retreatment, the median time until reintervention was 4.0 months (range 0.5-23.7 months). Of these seven patients, three had a repeat Optilume® dilatation, two had a bulbar urethroplasty, one had standard endoscopic dilatation and one restarted intermittent self-dilatation. There were no Clavian-Dindo ≥two complications.

There was no association with time to failure for age (HR 0.98, 95% CI 0.91-1.04, p=0.46), stricture length (HR 0.94, 95% CI 0.38-2.31, p=0.89), lumen size (HR 0.89, 95% CI 0.61-1.29, p=0.53) or number of prior treatments (HR 1.13, 95% CI 0.86-1.49, p=0.39) (Table 2). On Fisher’s exact test for subgroup analysis, there was no association with treatment failure for patients with idiopathic (p=0.10), iatrogenic (p=0.66), EBRT/BT (p=0.59) strictures, bladder neck contracture (p=0.34) or patients with diabetes (p=1.00) (Table 3). A meaningful subgroup analysis of other patient demographics, including vascular disease, was not possible due to low patient numbers.

**Table 2:** Hazard ratios demonstrating no association between time to failure with any stricture characteristic.

Stricture characteristic/patient demographic (n=38)	Hazard ratio	95% CI (lower)	95% CI (upper)	p value
Age	0.98	0.91	1.04	0.46
Number of previous stricture treatments	1.13	0.86	1.49	0.39
Stricture length	0.94	0.38	2.31	0.89
Stricture lumen size	0.89	0.61	1.29	0.53

**Table 3:** Fisher’s exact test demonstrating no association with failure for any stricture aetiology or patients with diabetes.

Stricture aetiology/patient demographic	p value
Idiopathic (n=17)	0.10
Iatrogenic (n=12)	0.66
Radiotherapy/brachytherapy induced (n=7)	0.59
Bladder neck contracture (n=2)	0.34
Diabetes (n=4)	1.00

## Discussion

To our knowledge, this is the first case series from the United Kingdom reporting outcomes of Optilume® balloon dilatation for recurrent strictures specifically aiming to identify stricture characteristics and patient factors that were associated with success. Overall, 82% patients in our series remained reintervention-free at a median follow-up of 22.9 months. At present, the National Institute of Clinical Excellence (NICE) precludes a recommendation on the use of Optilume® due to a paucity of long-term evidence on rates of reintervention and patient-reported outcome measures (PROMs) [14]. However, they recognise that Optilume® is significantly more cost-effective compared to standard endoscopic management, given the key factor in lowering treatment costs of urethral strictures is reducing the probability of recurrence [14,15]. Currently, NICE estimates the cost of a single Optilume® balloon to be £1350, excluding VAT [14].

Urethral stricture treatment outcomes are complicated to assess. They can be measured through anatomical patency, reintervention rates, uroflowmetry and/or PROMs. Most current published studies use a combination of these four parameters, although all published studies use reintervention-free rates due to its simplicity and its implications on both patient morbidity and healthcare costs [16-22]. Interestingly, our patient cohort was 82% reintervention-free at 22.9 months, which is similar to ROBUST III reintervention-free rates of 83% at one year and 78% at two years [22,23]. This is also comparable to other studies with similar follow-up times [17]. Uroflowmetry and PROMs were also found to improve and remain sustained in all published studies when included [13,16,23]. These were not included in our study due to both the difficulty of accessing uroflowmetry in a busy district general hospital, as well as the heterogeneity of timeframes at which patients returned their PROMs post Optilume®, meaning results were difficult to accurately interpret.

Another potential benefit of Optilume® is that it provides an effective option for patients unfit or unsuitable for urethroplasty. It is worth noting that in our series 11 (29%) patients had a significant co-morbidity and seven (19%) had prior pelvic radiation, making urethroplasty either high risk or technically very challenging. Interestingly, there was no association between time to failure for EBRT/BT-induced strictures, or any other aetiology or stricture characteristic, which is encouraging to all patients suffering with the morbidity of stricture disease. This is concurrent with ROBUST III results and Ballesteros et al., who found no predictors for stricture recurrence in a series of 156 patients treated with Optilume® [19,23]. Treatment with Optilume® has also been shown to be successful for patients with acute urinary retention secondary to stricture disease and those with prior urethroplasty [20,24]. Both patients in our series post urethroplasty have remained reintervention-free.

Of the seven patients in our series who required reintervention, the median time was 4.0 months, indicating that failure is likely to occur early. This could be considered encouraging for patients who may be suitable for urethroplasty, but wish to avoid its morbidity, as Optilume® treatment failure often declares itself early. Interestingly, of the seven patients who failed, three patients have had or opted for repeat Optilume® dilatation, demonstrating the perceived success of their earlier treatment.

The strengths of the study include its long-term follow-up, which is longer than most published series [19-21], and the wide heterogeneity of stricture aetiologies and patient demographics included. The primary use of reintervention rates will also help inform governing bodies, such as NICE, about its efficacy at reducing the risk of further treatment and therefore being more cost-effective. Its limitations include the small number of patients. Whilst it represents one of the larger case series, low overall numbers mean results, particularly within smaller subgroups, must be interpreted with caution. The study also did not look at uroflowmetry and PROMs as measures of success, which could help standardise both functional and patient satisfaction outcomes, something which NICE requires to help base further recommendations [14]. Finally, it is a single-centre retrospective study performed in a geographical region that has a predominantly Caucasian population [25].

## Conclusions

Optilume® balloon dilatation demonstrated excellent outcomes for recurrent bulbar urethral strictures across a wide range of stricture aetiologies, characteristics and patient demographics. It also provides an excellent option for patients who are unfit or wish to avoid the morbidity of urethroplasty. Although our study has small numbers, it represents one of the larger case series at present with a comparatively long length of follow-up. Larger and longer-term studies are required to validate our findings and to assess its efficacy as a potentially curative endoscopic treatment.
